# Lipidomic Analysis of Plasma Extracellular Vesicles Derived from Alzheimer’s Disease Patients

**DOI:** 10.3390/cells13080702

**Published:** 2024-04-18

**Authors:** Marios G. Krokidis, Krishna A. Pucha, Maja Mustapic, Themis P. Exarchos, Panagiotis Vlamos, Dimitrios Kapogiannis

**Affiliations:** 1 Laboratory of Bioinformatics and Human Electrophysiology, Department of Informatics, Ionian University, 49100 Corfu, Greece; mkrokidis@ionio.gr (M.G.K.); exarchos@ionio.gr (T.P.E.); vlamos@ionio.gr (P.V.); 2 Laboratory of Clinical Investigation, National Institute on Aging, National Institutes of Health (NIA/NIH), Baltimore, MD 21224, USA; ananthupucha@gmail.com (K.A.P.); maja.mustapi@nih.gov (M.M.)

**Keywords:** Alzheimer’s disease, extracellular vesicles, lipid profile, neurodegenerative diseases, biomarkers, exosomes

## Abstract

Analysis of blood-based indicators of brain health could provide an understanding of early disease mechanisms and pinpoint possible intervention strategies. By examining lipid profiles in extracellular vesicles (EVs), secreted particles from all cells, including astrocytes and neurons, and circulating in clinical samples, important insights regarding the brain’s composition can be gained. Herein, a targeted lipidomic analysis was carried out in EVs derived from plasma samples after removal of lipoproteins from individuals with Alzheimer’s disease (AD) and healthy controls. Differences were observed for selected lipid species of glycerolipids (GLs), glycerophospholipids (GPLs), lysophospholipids (LPLs) and sphingolipids (SLs) across three distinct EV subpopulations (all-cell origin, derived by immunocapture of CD9, CD81 and CD63; neuronal origin, derived by immunocapture of L1CAM; and astrocytic origin, derived by immunocapture of GLAST). The findings provide new insights into the lipid composition of EVs isolated from plasma samples regarding specific lipid families (MG, DG, Cer, PA, PC, PE, PI, LPI, LPE, LPC), as well as differences between AD and control individuals. This study emphasizes the crucial role of plasma EV lipidomics analysis as a comprehensive approach for identifying biomarkers and biological targets in AD and related disorders, facilitating early diagnosis and potentially informing novel interventions.

## 1. Introduction

Numerous studies indicate a link between Alzheimer’s (AD) and perturbed lipid metabolism, highlighting the pivotal role of lipids in the development of AD [1,2,3]. Plasma lipidomic analysis has been performed in mild cognitive impairment (MCI) and AD patients, showing that variations observed in lipid metabolites could signify transformations occurring in the neuronal membrane [4]. In a previous study, quantitative analysis of controls and patients with MCI and AD dementia indicated alterations in individual plasma lipid metabolites, such as 40 acylcantinites, 15 sphingolipids and 90 glycerophospholipids and altered levels of phosphocholine (PC) and lypoPC [5]. Another study identified differences in the plasma lipidome between AD patients and healthy age-matched controls for sphingomyelins, phosphatidylcholines, phosphatidylethanolamines, phosphatidylinositols and triglycerides, with a higher abundance of PC lipids in AD compared to control individuals, while more than 30% of lipids in ChE, PE and TG classes were linked with AD-related SNPs [6]. A recent study identified blood lipid modules enriched in sphingolipids, fatty acid pathways and diacylglycerols/phosphatidylethanolamines that were associated with brain volume measures, with some connections also found with amyloid-β (Aβ) and genetic risk for AD [7,8]. A targeted lipidomic exploration was performed in plasma samples from preclinical AD, MCI due to AD and healthy individuals and statistically significant alterations were detected for the diglycerol, lysophosphatidylethanolamine, lysophosphatidylcholine and sphingomyelin families, while 18:1 LPE showed statistically significant changes between preclinical/MCI-AD and healthy groups [9]. When it comes to cerebrospinal fluid, a study analyzed samples from cognitively healthy, MCI and late-onset AD participants showing a progressively diminution of 1-radyl-2-acyl- *sn* -glycerophosphocholine (PC), 1-radyl-2-acyl- *sn* -glycerophosphoethanolamine, and 1,2-diacyl- *sn* -glycerophosphoserine levels from cognitively healthy to MCI, and then to AD [8]. On the other hand, analysis of postmortem human brains of persons with mild AD, severe AD and cognitively normal controls showed alterations in different subgroups of lipids, such as glycerolipids, sphingolipids and glycerophosholipids, among them DAG 14:0/14:0, TAG 48:4/FA18:3, PE (p-18:0/18:1) and PS (18:1/18:2) [10]. The above highlights the apparent impossibility of establishing a consistent lipidomic expression pattern for AD, partly due to the heterogeneous origin of lipids in the plasma. This realization motivates the search for a more targeted starting material, specifically, extracellular vesicles (EVs) of brain cell origin, to enhance our understanding of lipidomic alterations in AD.

EVs are small (30–150 nm) membranous nanoparticles continually produced and secreted by all cells into their extracellular environment, playing vital roles in cellular homeostasis and inter-cellular communication. EVs are encircled by a phospholipid bilayer membrane that is enriched in cholesterol and ceramides. EVs are composed (mainly) of exosomes, vesicles that are generated within the late endosome by inward budding of the endosomal membrane, and microvesicles, which are produced by outward budding of the plasma membrane, thereby reflecting the composition of the respective membranes [11,12]. Cell-type-specific extracellular vehicles (EVs) have differential effects on the occurrence and progression of AD, such as participation in Aβ production and oligomerization as well as promotion of the clearance of toxic aggregates [13,14]. Recently, exploration of the lipid composition of brain-derived EVs from human postmortem frontal cortex tissues of AD patient and control subjects was performed, identifying significant changes in glycerophospholipids and sphingolipids levels, mainly in PE lipids and polyunsaturated fatty acid-containing lipids [15]. However, even this exploration was limited by the fact that brain-derived EVs have multiple cellular sources (neurons, glial cells) and by the singular origin of the frontal cortex, which may not reflect changes in other brain areas affected by AD.

EVs circulate in all biofluids, including blood, which contains EVs of diverse cellular origins, including of brain cellular origin. Different EV subpopulations carry cargos potentially revealing pathological processes in their cells of origin and may be used as biomarkers. Our group was one of the first that recognized the biomarker potential of neuronal-origin EVs in plasma for AD [16] and over the years, evidence has accumulated that EVs may provide diagnostic, prognostic and therapeutic response biomarkers for the disease [17]. Although previous studies focused on the EV proteome, characterization of district groups of lipids in different subfractions of EVs fractionated from human blood of AD patients may also be used as diagnostic or prognostic biomarkers due to the differential enrichment in phospholipids, ceramides and diacylglycerol levels [18]. However, apart from lipids constituting EVs, plasma lipoproteins co-precipitate along EVs. Plasma as such is a very challenging media for EV extraction. In another study, the ratio between lipid particles and EVs in plasma has been reported to be 10^12^ vs. 10^7^–10^9^ particles/mL, respectively [19]. There is scrutiny regarding EV isolation techniques, as most of them may co-precipitate lipoproteins, which have either comparable size or density. Karimi et al. reported a 100-fold decrease in lipid particle contamination by combining two isolating techniques [20]. Therefore, to perform lipidomic measurements in plasma that actually and accurately reflect EV composition, it is crucial to maximally deplete lipoproteins samples. To that end, first, we used immunoprecipitation as a means of negative selection to deplete ApoA1 and ApoE-containing lipoproteins, and, second, we used immunoprecipitation as a means of positive selection to capture three distinct EV subpopulations from plasma. Specifically, from the same plasma samples, we derived EVs of all-cell origin, derived by immunocapture of CD9, CD81 and CD63, transmembrane Tetraspanin proteins that are present (one or more of them) on all EVs; EVs of neuronal origin, derived by immunocapture of L1CAM; and EVs of astrocytic origin, derived by immunocapture of GLAST [21]. We sought to identify both AD-associated differences and differences by EV subtype to gain further insights in the identity of these EV subpopulations and the role of EV-associated lipids in AD pathogenesis.

## 2. Materials and Methods

### 2.1. Participants and Blood Draws

We analyzed plasma samples from 5 individuals with high-probability early Alzheimer’s disease (AD) (2 women, 3 men; age = (75.8 ± 4.5)) and 5 cognitively normal healthy controls (2 women, 3 men; age = (75.2 ± 4.9)). Diagnosis of high-probability early AD at the stage of mild cognitive impairment was based on the NIA-AA criteria [22] with typical clinical features and abnormal CSF levels of amyloid β-peptide (Aβ) 1–42 (Aβ42) < 192 pg/mL and of p181-tau > 23 pg/mL [23]. All participants were evaluated at the Clinical Research Unit of the U.S. National Institute on Aging (NIA; Baltimore, MD, USA) under NIH IRP-approved protocols. Blood was collected in 10 mL purple top (EDTA) tubes and was processed within 2 h by centrifugation at 2500× *g* for 15 min at room temperature (RT) to derive plasma. Plasma was collected, leaving 1 cm above the buffy coat, and spun again at 2500× *g*. Pre-analytical parameters affecting EV isolation and blood lipids (i.e., blood draws acquired in the morning after 12 h fasting, processing into EDTA plasma within 2 h and immediate aliquoting in cryo-vials, freezing in −80 °C, avoidance of additional thaws, slow thawing on ice on the day of processing into EVs) [24] were identical for samples of AD and control individuals.

### 2.2. Extracellular Vesicle Isolation

All samples were processed on the same day. Immediately after thawing, we added 5 thrombin to 0.5 mL plasma, mixed by inversion and incubated for 30 min. Afterwards, we centrifuged at 4000× *g* for 15 min, which generated defibrinated plasma that was filtered through a 0.22 μm filter and transferred to a fresh tube. We isolated total EVs using the Exoquick-LP Exosome Isolation kit (System Biosciences (SBI), Inc., Palo Alto, CA, USA), which includes a step of lipoprotein depletion by immunocapture. Following the manufacturer’s instructions, we conjugated magnetic beads separately with anti-ApoA1 (enriched in HDL) and anti-ApoE (enriched in LDL) and incubated with defibrinated plasma for 3 h at 4 °C. Following centrifugation and magnetic separation of the lipoprotein–bead complexes, lipoprotein-depleted plasma was transferred into clean tubes. Next, we proceeded with concentration of the remaining particles by adding Exoquick solution, mixing by inverting and subjecting to overnight incubation at 4 °C. The next day, the suspensions were centrifuged at 14,000 rpm, supernatants were removed and pellets containing crude EVs were resuspended in distilled water containing 1x protease and phosphatase inhibitors.

For each subject, we prepared three separate aliquots of 0.5 mL of lipoprotein-depleted crude EVs. To ensure homogeneity of the starting material, we pooled the three aliquots to 1.5 mL and re-aliquoted into clean tubes, each containing 0.5 ml. Each one of these re-aliquoted samples was then subjected to different immunoprecipitations, following the procedures described previously in detail [25]. Briefly, each 500 μL aliquot was incubated for 2 h at RT with 4 μg of biotinylated mouse anti-human CD171 (or else L1CAM) antibody (clone 5G3, Thermo Scientific, Inc., Waltham, MA, USA, # 13-1719-82), 4μg anti-GLAST (clone ACSA-1, Miltenyi Biotec, Auburn, CA, USA, #130-095-822) or a mix of antibodies against three Tetraspanins, 1.5 μg each (anti-CD81 [Ancell, Bayport, MN, USA, # 302-030], anti-CD63 [Abnova, Taipei City, Taiwan, #MAB15361] and anti-CD-9 [BD Pharmingen, San Diego, CA, USA, #558749]), followed by incubation with 40 μg of Pierce™ Streptavidin Plus UltraLink™ Resin (Thermo Scientific, Inc., Waltham, MA, USA) for 1 h at RT. After centrifugation, pellets were re-suspended in 200 μg of 0.1 M glycine–HCl to elute EV subpopulations followed by centrifugation to remove beads. Supernatants containing neuronal-enriched (L1CAM+) EVs, astrocytic-enriched (GLAST+) EVs or universal cellular origin (CD9+/CD63+/CD81+) EVs were transferred into clean tubes, and pH was neutralized with ammonium hydroxide. Samples were stored at −80 °C until shipment to Columbia University for lipidomic analysis. A portion of each sample was used to determine average EV concentration and diameter via nanoparticle tracking analysis (NTA) using Nanosight NS500 (Malvern Panalytical, Westborough, MA, USA) (Figure S1).

### 2.3. Targeted Lipidomics

Analysis was conducted at the Biomarkers Core Laboratory (BCL) of Columbia University. Briefly, lipidomics profiling was performed using ultra performance liquid chromatography–tandem mass spectrometry (UPLC-MSMS) [26,27]. Lipid extracts were prepared from samples spiked with appropriate internal standards using a modified Bligh and Dyer [28] method and analyzed on a platform comprising an Agilent 1260 Infinity HPLC integrated to an Agilent 6490A QQQ mass spectrometer controlled by Masshunter v 7.0 (Agilent Technologies, Santa Clara, CA, USA). Glycerophospholipids and sphingolipids were separated with normal-phase HPLC as described before [29], with a few modifications. An Agilent Zorbax Rx-Sil column (2.1 × 100 mm, 1.8 µm) maintained at 25 °C was used under the following conditions: mobile phase A (chloroform: methanol: ammonium hydroxide, 89.9:10:0.1, *v*/*v*) and mobile phase B (chloroform: methanol: water: ammonium hydroxide, 55:39:5.9:0.1, *v*/*v*); 95% A for 2 min, decreased linearly to 30% A over 18 min and further decreased to 25% A over 3 min, before returning to 95% over 2 min and held for 6 min. Separation of sterols and glycerolipids was carried out on a reverse-phase Agilent Zorbax Eclipse XDB-C18 column (4.6 × 100 mm, 3.5 μm) using an isocratic mobile phase, chloroform, methanol, 0.1 M ammonium acetate (25:25:1) at a flow rate of 300 μL/min.

Quantification of lipid species was accomplished using multiple reaction monitoring (MRM) transitions under both positive and negative ionization modes in conjunction with referencing of appropriate internal standards: PA 14:0/14:0, PC 14:0/14:0, PE 14:0/14:0, PG 15:0/15:0, PI 17:0/20:4, PS 14:0/14:0, BMP 14:0/14:0, APG 14:0/14:0, LPC 17:0, LPE 14:0, LPI 13:0, Cer d18:1/17:0, SM d18:1/12:0, dhSM d18:0/12:0, GalCer d18:1/12:0, GluCer d18:1/12:0, LacCer d18:1/12:0, D7-cholesterol, CE 17:0, MG 17:0, 4ME 16:0 diether DG, D5-TG 16:0/18:0/16:0 (Avanti Polar Lipids, Alabaster, AL, USA). Lipid levels for each sample were calculated by summing up the total number of moles of all lipid species measured by all three LC-MS methodologies and then normalizing that total to mol %.

### 2.4. Statistical Methods

**Figure 5:**
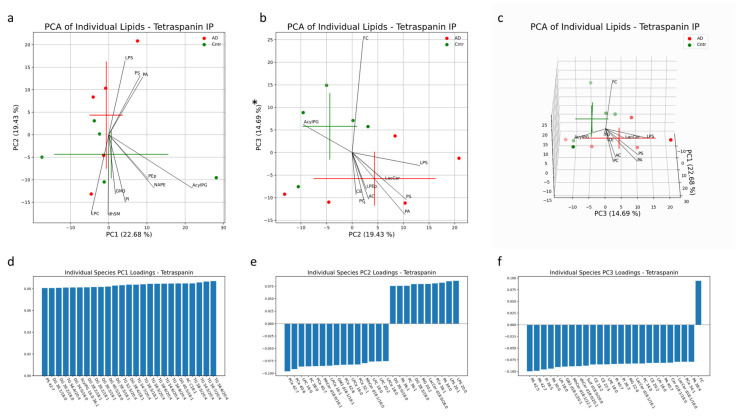
Multivariate analysis was conducted on individual lipid molecules that were detected via MS in EV samples isolated with a pan-Tetraspanin IP. Crosses indicate the center of the subject-group cluster, with the vertical and horizontal components representing the standard deviation of a group along the corresponding PC (**a**–**c**). Asterisks indicate a significant difference (*p* < 0.05) between AD and control groups along a PC. Black lines represent the magnitude and direction of the pooled lipid species’ relative contribution to any separation between AD and control clusters. The top 10 most influential lipid species are depicted. The 25 largest individual lipid contributions to variability along principal components 1, 2 and 3 are depicted through loadings plots (**d**–**f**).

**Figure 6:**
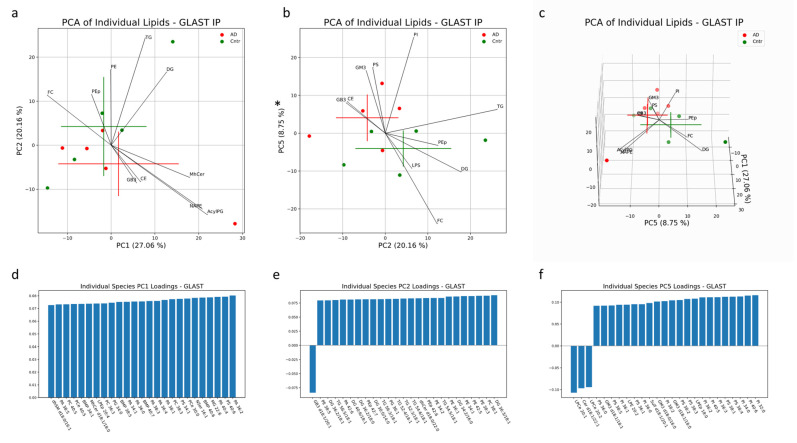
Multivariate analysis was conducted on individual lipid molecules that were detected via MS in EV samples isolated with a GLAST IP. Crosses indicate the center of the subject-group cluster, with the vertical and horizontal components representing the standard deviation of a group along the corresponding PC (**a**–**c**). Asterisks indicate a significant difference (*p* < 0.05) between AD and control groups along a PC. Black lines represent the magnitude and direction of the pooled lipid species’ relative contribution to any separation between AD and control clusters. The top 10 most influential lipid species are depicted. The 25 largest individual lipid contributions to variability along principal components 1, 2 and 5 are depicted through loadings plots (**d**–**f**).

**Figure 7:**
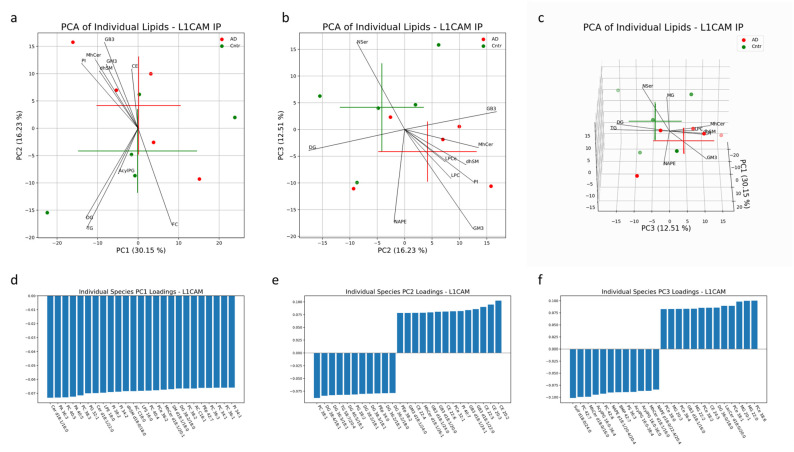
Multivariate analysis was conducted on individual lipid molecules that were detected via MS in EV samples isolated with an L1CAM IP. Crosses indicate the center of the subject-group cluster, with the vertical and horizontal components representing the standard deviation of a group along the corresponding PC (**a**–**c**). Black lines represent the magnitude and direction of the pooled lipid species’ relative contribution to any separation between AD and control clusters. The top 10 most influential lipid species are depicted. The 25 largest individual lipid contributions to variability along principal components 1, 2 and 3 are depicted through loadings plots (**d**–**f**).

**Figure 8:**
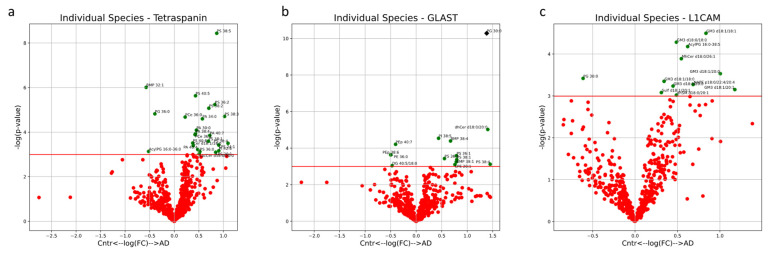
Volcano plots describe the magnitude and significance of differences in individual lipid concentrations between AD and control EV samples. EVs were isolated with pan-Tetraspanin (**a**), GLAST (**b**) or L1CAM (**c**) IP. Green points above the horizontal red line depict lipids with concentrations significantly different (*p* < 0.05) between AD and control samples. Black diamonds above the horizontal red line depict lipids with concentrations significantly different (*p* < 0.05) after multiple-testing (Bonferroni) correction between AD and control samples. Positive values on the horizontal axis indicate enrichment in AD samples and vice versa.

**Figure 9:**
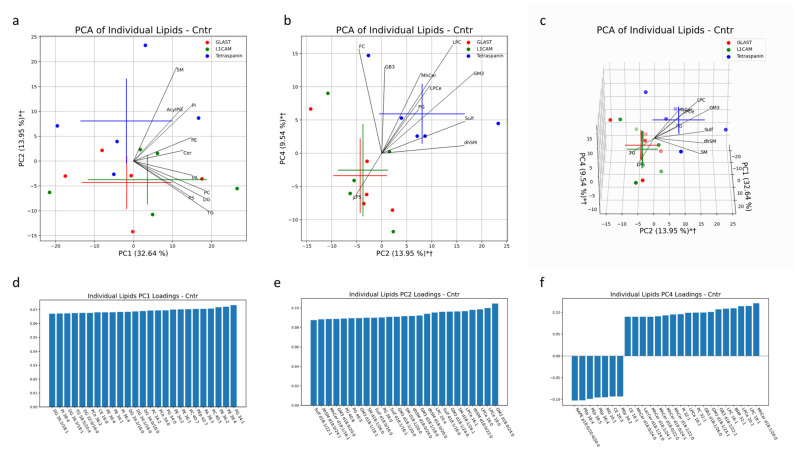
Multivariate analysis was conducted on individual lipid molecules that were detected via MS in EV samples from healthy human subjects. Crosses indicate the center of an EV subset cluster, with the vertical and horizontal components representing the standard deviation of a group along the corresponding PC (**a**–**c**). * indicates a significant difference (*p* < 0.05) between Tetraspanin IP EVs and L1CAM IP EVs along a PC. † indicates a significant difference (*p* < 0.05) between Tetraspanin IP EVs and GLAST IP EVs along a PC. Black lines represent the magnitude and direction of the pooled lipid species’ relative contribution to any separation between pan-EV (Tetraspanin IP EVs) and brain-associated EVs (L1CAM and GLAST IP EVs). The top 10 most influential lipid species are depicted. The 25 largest individual lipid contributions to variability along principal components 1, 2 and 4 are depicted through loadings plots (**d**–**f**).

**Figure 10:**
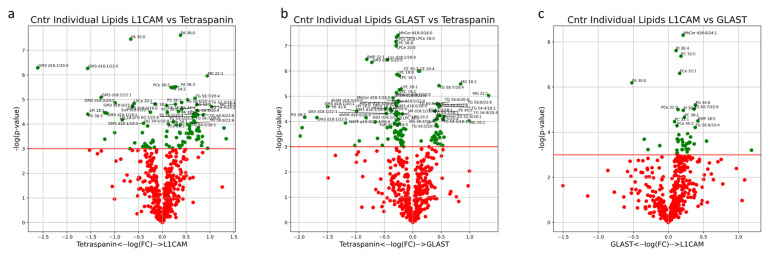
Volcano plots describe the magnitude and significance of differences in individual lipid concentrations between EV subsets in healthy control subjects (L1CAM vs. Tetraspanin (**a**), GLAST vs. Tetraspanin (**b**), L1CAM vs. GLAST (**c**)). EVs were isolated with L1CAM, GLAST or pan-Tetraspanin IP. Green points above the horizontal red line depict lipids with concentrations significantly different (*p* < 0.05) between AD and control samples. Black diamonds above the horizontal red line depict lipids with concentrations significantly different (*p* < 0.05) after multiple-testing (Bonferroni) correction between AD and control samples.

**Figure 11:**
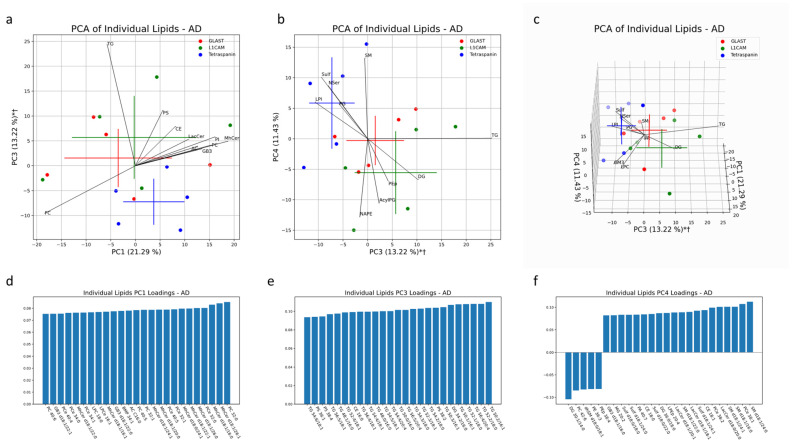
Multivariate analysis was conducted on individual lipid molecules that were detected via MS in EV samples from AD patients. Crosses indicate the center of an EV subset cluster, with the vertical and horizontal components representing the standard deviation of a group along the corresponding PC (**a**–**c**). * indicates a significant difference (*p* < 0.05) between Tetraspanin IP EVs and L1CAM IP EVs along a PC. † indicates a significant difference (*p* < 0.05) between Tetraspanin IP EVs and GLAST IP EVs along a PC. Black lines represent the magnitude and direction of the pooled lipid species’ relative contribution to any separation between pan-EV (Tetraspanin IP EVs) and brain-associated EVs (L1CAM and GLAST IP EVs). The top 10 most influential lipid species are depicted. The 25 largest individual lipid contributions to variability along principal components 1, 3 and 4 are depicted through loadings plots (**d**–**f**).

**Figure 12:**
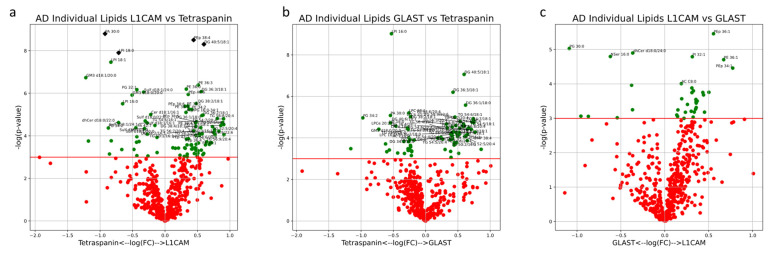
Volcano plots describe the magnitude and significance of differences in individual lipid concentrations between EV subsets in AD patients (L1CAM vs. Tetraspanin (**a**), GLAST vs. Tetraspanin (**b**), L1CAM vs. GLAST (**c**)). EVs were isolated with L1CAM, GLAST or pan-Tetraspanin IP. Green points above the horizontal red line depict lipids with concentrations significantly different (*p* < 0.05) between AD and control samples. Black diamonds above the horizontal red line depict lipids with concentrations significantly different (*p* < 0.05) after multiple-testing (Bonferroni) correction between AD and control samples.

The data were expressed as the mean value ± standard deviation (SD) of lipid classes from the measurement of five samples per group. Statistical significance of the results was calculated by unpaired two-tailed Student’s *t*-test using GraphPad Prism™ software version 6.01 for Windows (GraphPad Software Inc., La Jolla, CA, USA). In addition to the main analysis presented above, we performed a multivariate analysis to replicate and increase our confidence in univariate results. For these analyses, percent total lipid composition values were further normalized by log-transformed EV particle concentrations by NTA. Multivariate principal component analysis (PCA) and follow-up univariate analyses were performed on the processed data. Natural logarithmic-transformed fold changes in lipid concentrations between AD and control groups vs. negative natural logarithmic-transformed *p*-values are depicted in the volcano plots (Figure 8, Figure 10 and Figure 12). Comparisons between AD and control samples were made within each EV subtype. Additionally, the three EV subtypes were independently compared to each other within each patient group (healthy controls or AD). A standard scaler was applied to the input data before PCA was performed. PCA is an unsupervised dimension reduction methodology that highlights the axes, or principal components (PC), that account for the greatest variance between samples. PCs that best depicted separation between clusters were chosen to be visualized in the PCA plots (Figure 5a–c, Figure 6a–c, Figure 7a–c, Figure 9a–c and Figure 11a–c). Loading vectors of the individual molecules from each lipid species were averaged and compared to the vector describing the relative direction of the AD cluster compared to the control cluster (separation vector) (Figure 5a–c, Figure 6a–c and Figure 7a–c). The separation vector for the EV subtype characterization was calculated as the vector describing the relative position of the all-cell-origin EV cluster compared to the two brain-related cell-type-derived EV clusters (Figure 9a–c and Figure 11a–c). The average loading vector of each species was then projected on the separation vector. The magnitude and direction of influence for species with relatively high projection vector magnitudes (top 10) are displayed in the corresponding PC plots (Figure 5a–c, Figure 6a–c, Figure 7a–c, Figure 9a–c and Figure 11a–c). Individual lipids with large absolute loadings (top 25) are also presented (Figure 5d–f, Figure 6d–f, Figure 7d–f, Figure 9d–f and Figure 11d–f).

## 3. Results

There were no differences in EV average concentration of NEVs, AEVs, or multi-origin EVs between AD and healthy control (HC) individuals. HC samples contained a lower concentration of AEVs compared to NEVs, a difference not seen for AD samples. Moreover, NEVs of AD individuals had a higher-diameter mode compared to that of NEVS of HC (Figure S2). Targeted lipidomics was performed on the three EV fractions, allowing the quantification of 593 lipid species, including lipid classes from the sterol lipid, glycerolipid, sphingolipid, glycerophospholipid and lysophospholipid categories (Table S1). The relative composition of EVs in the identified lipid categories expressed as normalized relative abundance differed across groups (AD and control individuals) and EV classes (Table 1). We observed a significant enhancement in glycerophospholipid abundance in neuronal EVs (NEVs, marked as L1CAM) compared to multi-origin EVs (marked as Tetraspanin-EVs) in control samples (*p* = 0.023), as well as in the lysophospholipids pool between astrocytic EVs (AEVs, marked as GLAST) and multi-origin EVs in both AD and control samples (*p* = 0.021 and *p* = 0.005, respectively).

**Figure 1:**
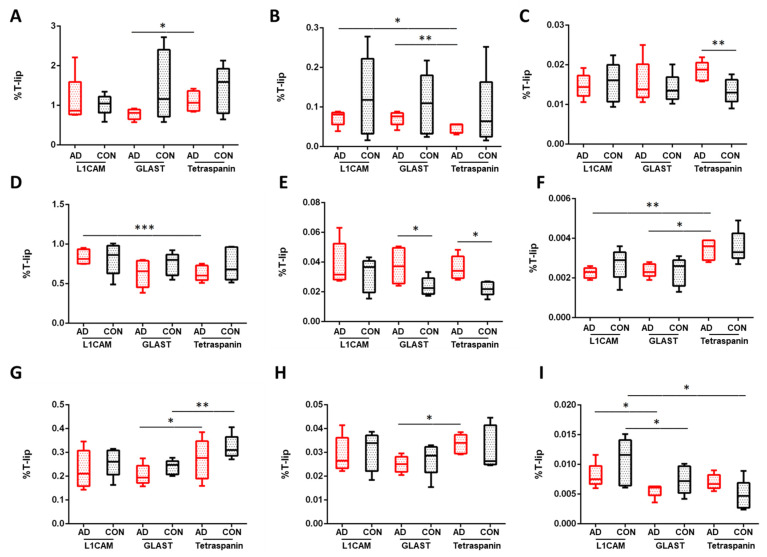
Percentage differences for representative classes of glycerolipids (GLs), glycerophospholipids (GPLs) and lysophospholipids (LPLs) in EV subtypes of AD and control samples. (**A**) Monoacylglycerol, (**B**) diacylglycerol, (**C**) phosphatidic acid, (**D**) phosphatidylethanolamine, (**E**) phosphatidylserine, (**F**) phosphatidylglycerol, (**G**) lysophosphatidylcholine, (**H**) lysophosphatidylethanolamine and (**I**) lysophosphatidylserine lipids. L1CAM are neuronal EVs; GLAST are astrocytes EVs; Tetraspanin-EVs are multi-origin EVs (CD81, CD9, CDC3; surrogate of all-cell origin). The values are given as mean ± SD (*n* = 5). Statistical significance: * (*p* < 0.05), ** (*p* < 0.01), *** (*p* < 0.001). For specific values, see Table S1.

Comparing the specific lipid composition of each class of EVs, profound differences were detected, as Figure 1 depicts (see Tables S1 and S2 for detailed values).

### 3.1. Neuronal EVs

Focusing on sphingolipids, dihydroceramide (dhCer) levels were significantly decreased in NEVs compared to AEVs (*p* = 0.008) in AD samples, while enhanced levels of monohexosylceramide (MhCer) were observed between samples of controls (*p* = 0.007). The analysis showed limited alterations in glycerolipid levels among NEVs and multi-origin EVs families, as Figure 1 depicts (see Tables S1 and S2 for detailed values). Increased levels of diacylglycerol were seen in NEVs from AD samples compared to multi-origin EVs (*p* = 0.015, Figure 1B). No differences were found in triacylglycerol levels between control and AD NEVs (Figure S2). Raised levels of sulfatides were also indicated between multi-origin EVs and NEVs derived from AD patients (*p* = 0.031), as well as in controls (*p* = 0.006). Lastly, significantly decreased levels of GM3 were depicted in NEVs of controls compared to multi-origin EV samples (*p* = 0.013). No differences were found in ceramide, sphingomyelin and lactoceramide levels between control and AD samples (Tables S1 and S2). Among glycerophospholipids (Figure 1, total GLP classes in Figure S3 and Table S1), higher levels of phosphatidylcholine (PC) were found for control NEVs compared to AEVs (*p* = 0.041) and multi-origin EVs (*p* = 0.017). Significantly raised levels of phosphatidylethanolamine (PE) and plasmalogen phosphatidylethanolamine (PEp) were also observed in NEVs derived from AD patients compared to multi-origin EV samples (*p* = 0.0006 for PE; *p* = 0.002 for Pep). On the other hand, phosphatidylglycerol (PG) was significantly lower in NEVs compared to multi-origin EV samples (*p* = 0.005 Figure 1F). Lysophospholipids (LPLs), deacylated forms of phospholipids with a single fatty acid chain, were also measured, with an enhancement of lysophosphatidycholine (LPC) and lysophosphatidylethanolamine (LPE) in multi-origin EVs compared to NEVs of AD patients (*p* = 0.038, Figure 1G and *p* = 0.032, Figure 1H). Furthermore, significantly lower levels of lysophosphatidylserine (LPS) were observed in NEVs compared to AEVs in AD (*p* = 0.032. Figure 1I).

### 3.2. Astrocytic EVs

Phosphatidylserine (PS) was increased in AEVs derived from AD compared to control samples (*p* = 0.017, Figure 1, Table S1). The analysis also showed significantly lower levels of phosphatidylglycerol (PG) in AEVs compared to multi-origin EVs in AD samples (*p* = 0.024; Figure 1, Table S1). Lysophosphatidylcholine (LPC), a glycerophospholipid derived from the hydrolysis of one fatty acid ester of PC by phospholipase A_2_, was significantly reduced in AEVs compared to multi-origin EVs in AD and control samples (*p* = 0.038 and *p* = 0.003, respectively, Figure 1 and Table S1), as well as ether lysophosphatidylcholine (LPCs) (*p* = 0.032 and *p* = 0.0009, respectively, Figure S4 and Table S1). Moreover, a diminution of lysophosphatidylethanolamine (LPE) was found in AEVs derived from AD samples compared to multi-origin EVs (*p* = 0.032, Figure 1 and Table S1), while lower levels of LPS were indicated in AEVs compared to NEVs (*p* = 0.032 in AD, *p* = 0.026 in controls). Significantly higher levels of LPI were observed in multi-origin EVs of AD samples compared to NEVs (*p* = 0.002) and astrocytes EVs (*p* = 0.002), as well as multi-origin EVs of control samples (*p* = 0.006 for neuronal EVs and *p* = 0.004 for astrocytic EVs), as Figure S4 shows. Among glycerolipids, monoacylglycerol levels were significantly higher in AEVs derived from AD patients compared to multi-origin EVs (*p* = 0.026), whereas a decrease in diacylglycerol levels was also seen between these EV types (*p* = 0.009), as Figure 1 shows. No differences were found in triacylglycerol levels between control and AD samples (Figure S2). Furthermore, specific classes of sphingolipids such as dihydrosphingomyelin, monohexosyl ceramidewere, sulfatide, GM3 and GB3 were diminished in control AEVs compared to multi-origin EVs (*p* = 0.045 for dhSM; 0.023 for MhCer; 0.032 for Sulf; 0.008 for GM3; 0.021 for GB3), without significant alterations between EV types in AD samples (Figure S2 and Tables S1 and S2). Lastly, important insights were gained for the comparison between AEVs and multi-origin EVs controls, such as a diminution of bis(monoacylglycero)phosphate (BMP) (*p* = 0.048), an enhancement of free cholesterol (*p* = 0.023), as well as a decrease in cholesterol ester levels (*p* = 0.027) (Tables S1 and S2).

### 3.3. Multi-Origin EVs

Two important glycerophospholipids, phosphatidic acid (PA) and phosphatidylserine (PS), were increased in multi-origin EVs of AD patients compared to control samples (*p* = 0.003 and *p* = 0.021, respectively, Figure 1, Table S1). All the other significant changes between multi-origin EVs and NEVs or AEVs have been extensively described above.

### 3.4. Cholesterol Ester and Glycerolipid Levels in EV Cargo

**Figure 2:**
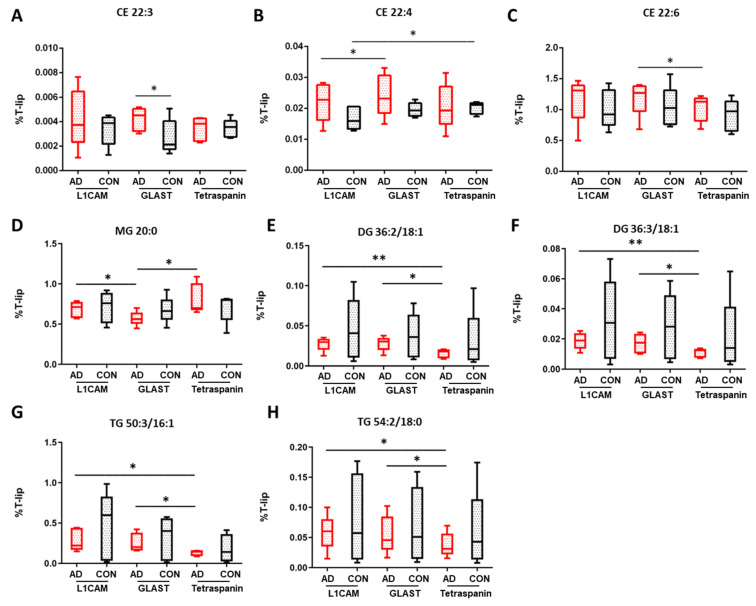
Percentage differences for selected cholesterol esters (CE) and glycerolipid species in EV subtypes of AD and control samples. (**A**) CE 22:3, (**B**) CE 22:4, (**C**) CE 22:6, (**D**) MG 20:0, (**E**) DG 36:2/18:1, (**F**) DG 36:3/18:1, (**G**) TG 50:3/16:1, (**H**) TG 54:2/18:0. L1CAM are neuronal EVs; GLAST are astrocyte EVs; Tetraspanin-EVs are multi-origin EVs (CD81, CD9, CDC3, surrogate of all-cell origin). The values are given as mean ± SD (*n* = 5). Statistical significance: * (*p* < 0.05), ** (*p* < 0.01). For specific values, see Table S3.

Τhe most highly abundant lipid class detected was cholesterol esters (CE), with some of their groups presenting significant differences between EV types (Table S3). As shown in Figure 2, an increase in CE 22:3 was observed in AEVs derived from AD patients compared to multi-origin EVs (*p* = 0.027), in CE 22:4 between AD AEVs and NEVs (*p* = 0.042), as well as in CE 22:6 abundance between AEVs and multi-origin EVs in AD samples (*p* = 0.041). A diminution of CE 20:3 was found in control multi-origin EVs compared to control AEVs (*p* = 0.001), as well as in control NEVs compared to multi-origin EVs for CE 22:4 species (*p* = 0.028). CE 18:2 and CE 20:4 were the most abundant among CE lipids (Table S3).

Comparing individual glycerolipid classes in EVs, important insights were generated. Of the detected monoacylglycerol species, there was a larger proportion of saturated fatty acids (SFA) compared to monounsaturated fatty acids or polyunsaturated fatty acids (PUFA). The three detected SFA species MG 16:0, MG 18:0 and MG 20:0 presented the highest overall relative abundance in all EV types (Table S3). MG 20:0 was decreased in AD AEVs compared to NEVs (*p* = 0.024) and multi-origin EVs (*p* = 0.033), as Figure 2 shows. MG 20:3 and MG 22:2 levels were significantly higher in multi-origin EVs in AD compared to control samples (*p* = 0.013 and *p* = 0.020, respectively, Table S3). Significant differences were also found in MG 18:0, MG 18:2 and MG 20:1 levels between control NEV and multi-origin EV samples (*p* = 0.047, *p* = 0.044 and *p* = 0.048), as well as in MG 18:2, MG 20:1, MG 20:2 and MG 22:1 between control AEV and multi-origin EVs (*p* = 0.001, *p* = 0.030, *p* = 0.041, *p* = 0.007). Focusing on diacylglycerol lipids, significantly altered species were detected for AD NEVs and AEVs compared to multi-origin EVs, such as DG 36:2/18:1 (*p* = 0.009 and *p* = 0.012, Figure 2E) and DG 36:3/18:1 (*p* = 0.006 and *p* = 0.021, Figure 2F). Significant changes in the contents of DG species were also observed for these EV types when comparing AD and control samples (34:2/16:0, 34:2/16:1, 36:1/18:0, 38:2/18:1, 38:4/18:1, 40:5/18:1 and 40:6/18:1). Lower levels of DG 40:5/18:0 were found in AEVs of AD compared to control samples (*p* = 0.044). In triacylglycerol lipids, significant alterations were detected in neuronal and astrocytic EVs compared to multi-origin EVs, such as TG 50:3/16:1 (*p* = 0.042 and *p* = 0.045, respectively) and TG 54:2/18:0 (*p* = 0.024 and *p* = 0.034, respectively), as shown in Figure 2G,H. Similar trends were observed in TG species containing MUFA (52:5, 54:6, 54:7, 56:3) and omega-6 PUFA (54:5, 54:6, 54:7, 56:4, 56:7, 56:8, 56:9, 58:5, 58:6, Table S3) while significant differences were noted for lipid species incorporating omega-6 PUFA such as TG 60:7/22:6 and TG 60:9/22:6 between neuronal AD and control samples (*p* = 0.009 and *p* = 0.036, respectively).

### 3.5. Sphingolipid, Glycerophospholipid and Lysophosholipid Levels in EVs

Shingomyelin (SM) species were the most enriched class of sphingolipids detected in EVs, with SM d18:1/16:0 and SM d18:1/16:1 being significantly reduced in neuronal EVs compared to multi-origin EVs in AD samples (*p* = 0.030 and *p* = 0.009, Table S3). Dihydrosphingomyelin (dhSM) species also followed suit, with diminished levels of dhSMd18:0/16:0 found in neuronal EVs compared to multi-origin EVs in AD samples (*p* = 0.024), while most of dhSM lipids were significantly altered between astrocytic EVs and multi-origin EVs of control samples (Table S3). Similarly, lower levels of Cer d18:1/16:1, Cer d18:1/20:1 and Cer d18:1/24:1 were observed between NEVs compared to multi-origin EVs of AD samples (*p* = 0.012, *p* = 0.046, *p* = 0.074, Table S3), as well as for Cer d18:1/16:0 levels in neuronal and astrocytic EVs compared to multi-origin EVs of control samples (*p* = 0.010 and *p* = 0.007, respectively). Moreover, significant changes in the contents of dihydroceramide (dhCer) molecular species were observed when comparing neuronal and astrocytic EVs in AD (d18:0/20:0, *p* = 0.036; d18:0/22:1, *p* = 0.048 and d18:0/24:0, *p* = 0.005), as well as neuronal EVs, astrocytic EVs and multi-origin EVs, respectively (d18:0/22:0, *p* = 0.024 and d18:0/18:1, *p* = 0.047), as presented in Table S3. Focusing on sulfatide lipids, significantly altered species were identified among AD neuronal and astrocytic EVs compared to CD subfamilies (Table S3) such as d18:0/22:0 (*p* = 0.010 and *p* = 0.027), d18:1/16:1 (*p* = 0.020 and *p* = 0.030) and d18:1/20:0 (*p* = 0.015 and *p* = 0.030). Sulf d18:1/18:0 was decreased in neuronal EVs compared to multi-origin EVs of AD samples (*p* = 0.010), along with d18:1/24:0 (*p* = 0.004) and d18:1/24:1 (*p* = 0.015). Both species were also significantly altered in control samples (*p* = 0.010 and 0.013, respectively).

**Figure 3:**
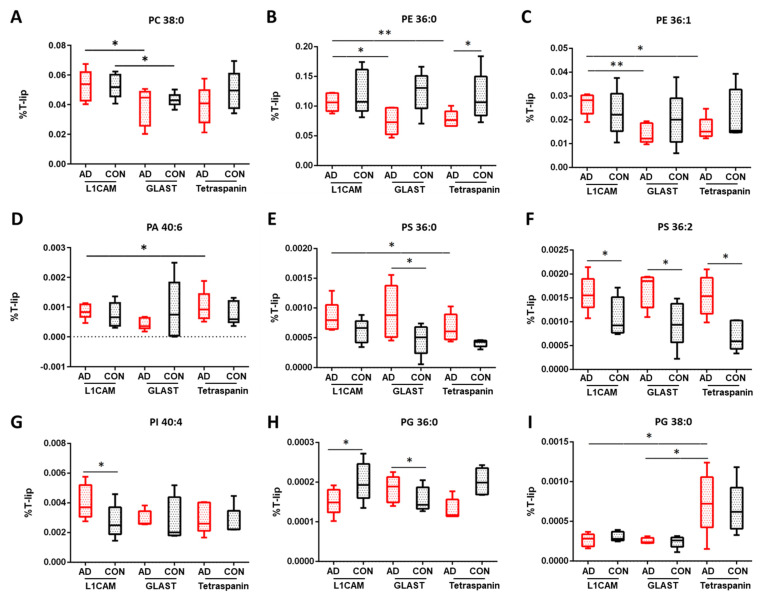
Percentage differences for selected glycerophospholipid species in EV subtypes of AD and control samples. (**A**) PC 38:0, (**B**) PE 36:0, (**C**) PE 36:1, (**D**) PA 40:6, (**E**) PS 36:0, (**F**) PS 36:1, (**G**) PI 40:4, (**H**) PG 36:0, (**I**) PG 38:0. L1CAM are neuronal EVs; GLAST are astrocyte EVs; Tetraspanin-EVs are multi-origin EVs (CD81, CD9, CDC3, surrogate of all-cell origin). The values are given as mean ± SD (*n* = 5). Statistical significance: * (*p* < 0.05), ** (*p* < 0.01). For specific values, see Table S3.

Focusing on individual glycerophospholipids (GLPs) classes in EVs, important differences were observed between AD and control samples, as Figure 3 shows. Phosphatidylcholine (PC) and phosphatidylethanolamine (PE) species were the most enriched classes of GLPs detected in EVs (Figure S4, Supplementary Materials). Specifically, significant changes in the contents of PC species were seen when comparing AD neuronal and astrocytic EVs (32:1, *p* = 0.025; 38:0, *p* = 0.015; 40:7, *p* = 0.016; 42:7, *p* = 0.039), AD neuronal Evs and multi-origin Evs (38:1, *p* = 0.038; 40:7, *p* = 0.040; 42:7, *p* = 0.043) and multi-origin Evs between AD and control samples (34:0, *p* = 0.033; 34:1, *p* = 0.041; 36:0, *p* = 0.044, 36:1, *p* = 0.036; 389:3, *p* = 0.040), as shown in Table S3. We also proceeded with the exploration of ether PC species (Table S3). Reduced levels of PCe 38:1 and PCe 40:7 in neuronal compared to astrocytic EVs in AD samples (*p* = 0.049 and *p* = 0.018, respectively), in AD neuronal EVs compared to multi-origin EVs (PCe 36:0, *p* = 0.042 and PCe 36:3, *p* = 0.049) and in AD astrocytes EVs compared to multi-origin EVs (PCe 32:0, *p* = 0.016 and PCe 36:0, *p* = 0.024).

In terms of PE lipids, significant alterations were found in astrocytic EVs between AD and control samples (36:0, *p* = 0.026 and 38:0, *p* = 0.030, Table S3), as well as in AD samples comparing (i) neuronal and astrocytic EVs (36:0, *p* = 0.050; 36:1, *p* = 0.008; 38:1, *p* = 0.041), (ii) neuronal and multi-origin EVs (34:2, *p* = 0.014; 36:0, *p* = 0.001; 36:1, *p* = 0.020; 36:3, *p* = 0.007; 38:0, *p* = 0.006; 38:1, *p* = 0.010, 38:3, *p* = 0.039; 38:4, *p* = 0.010) and (iii) astrocytic and multi-origin EVs (32:, *p* = 0.026). In control samples, increased levels of PE 32:0 and PE 36:4 were found between neuronal and astrocytic EVs (*p* = 0.041 and *p* = 0.020, respectively), an enhancement of PE 38:1 when comparing neuronal and multi-origin EVs (*p* = 0.005) and a diminution of PE 32:0, PE 40:5 and PE 42:6 when comparing astrocytic and multi-origin EVs (*p* = 0.026, *p* = 0.008 and *p* = 0.027, respectively, Table S3). The analysis of plasmalogen phosphatidylethanolaminelipids (Pep), an important class of GLPs, which contain a characteristic vinyl ether double bond, showed significant alterations in AD samples when comparing neuronal and astrocytic EVs (34:1, *p* = 0.005; 38:6, *p* = 0.013, 40:7, *p* = 0.040) as well as neuronal and multi-origin EVs (34:1, *p* = 0.027, 34:2, *p* = 0.036, 38:4, *p* = 0.0004, 38:5, *p* = 0.019, 38:6, *p* = 0.002, 40:6, *p* = 0.004; Table S3). Focusing on phosphatidic acid (PA) contents (Table S3), raised levels of PA 30:0 were seen when comparing AD neuronal and astrocytic EVs (*p* = 0.008), as well as in multi-origin EVs compared to neuronal (*p* = 0.004) and astrocytic EVs (*p* = 0.006). Moreover, a diminution in PA 40:6 was observed when comparing neuronal and astrocytic EVs (*p* = 0.044) and astrocytic EVs and multi-origin EVs (*p* = 0.010) in AD samples. Significant differences were also found between AD and control multi-origin EVs (Table S3). On the other hand, regarding phoshatidylserine (PS) lipids (Table S3), an enhancement of 36:2 and 38:2 species was seen when comparing AD neuronal EVs compared to controls (*p* = 0.038 and *p* = 0.029, respectively). Furthermore, differences were also seen for astrocytic EVs derived from AD samples compared to controls (36:0, *p* = 0.041; 36:1, *p* = 0.038; 36:2, *p* = 0.024; 38:1, *p* = 0.032; 38:2, *p* = 0.039, 42:7, *p* = 0.017), as well as when comparing multi-origin EVs between AD and control samples, such as 36:2 (*p* = 0.039), 38:45 (*p* = 0.003) and 40:6 (*p* = 0.014). Limited alterations were found in the contents of phoshatidylinositol (PI) molecular species (Table S3), such as a difference in PI 40:4 in neuronal EVs when comparing AD and controls samples (*p* = 0.027, Figure 3G) and PI 36:2 when comparing neuronal EVs and multi-origin EVs in AD samples (*p* = 0.032). Regarding phoshatidyglycerol (PG) lipids, significant alterations were observed in the levels of PG 30:0 and PG 36:0 when comparing AD and control samples of astrocytic EVs (*p* = 0.0001 and *p* = 0.045, respectively), as well as when comparing AD and control samples of neuronal EVs (*p* = 0.005) for PG 30:0 species. The observed changes in AD samples should also be stressed here when comparing (i) neuronal and astrocytic EVs (PG 30:0, *p* = 0.001; PG 36:3, *p* = 0.036; PG 38:1, *p* = 0.049), (ii) neuronal EVs and multi-origin EVs (PG 36:1, *p* = 0.010, PG 36:4, *p* = 0.012, PG 38:3, *p* = 0.022, PG 40:6, *p* = 0.033) and (iii) astrocytic EVs and multi-origin EVs (PG 34:2, *p* = 0.013, PG 38:0, *p* = 0.045, PG 40:6, *p* = 0.013), as presented in Figure 3 and shown in Table S3.

**Figure 4:**
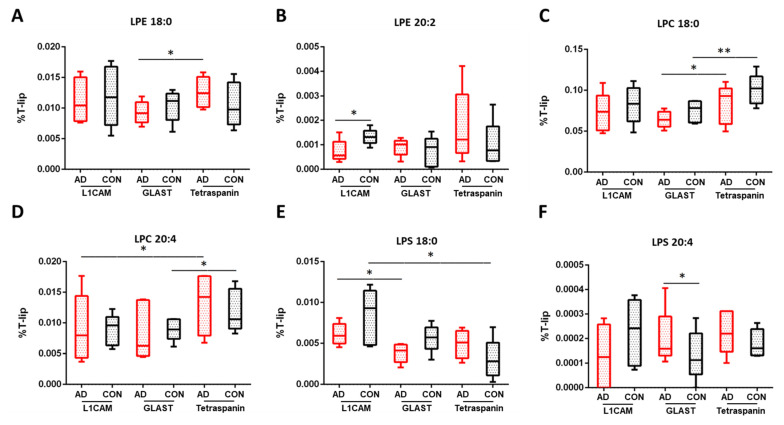
Percentage differences for selected lysophospholipid species in EV subtypes of AD and control samples. (**A**) LPE 180, (**B**) LPE 20:2, (**C**) LPC 18:0, (**D**) LPC 20:4, (**E**) LPS 18:0, (**F**) LPS 20:4. L1CAM are neuronal EVs; GLAST are astrocyte EVs; Tetraspanin-EVs are multi-origin EVs (CD81, CD9, CDC3, surrogate of all-cell origin). The values are given as mean ± SD (*n* = 5). Statistical significance: * (*p* < 0.05), ** (*p* < 0.01). For specific values, see Table S3.

Lastly, important differences were observed for lysophospholipids (LPLs), such as fold differences in individual LPC, LPE, LPI and LPS species. Significant changes were found in lysophosphatidylethanolamine (LPE) molecular species when comparing AD and control neuronal (20:2, *p* = 0.015) and astrocytic (20:1, *p* = 0.011) EVs (Table S3). Furthermore, reduced levels of 20:3 were observed in AD neuronal EVs compared to astrocytic ones (*p* = 0.045), as well as for 18:0 in AD astrocytic EVs compared to multi-origin EVs (*p* = 0.080, Figure 4A). Most lysophosphatidylcholine (LPC) lipids were significantly altered when comparing astrocytic EVs and to multi-origin EVs in AD and control samples (16:1, 18:0, 18:1, 20:1, 20:2), as well as 20:3 and 20:4 when comparing AD neuronal EVs and multi-origin EVs (*p* = 0.044 and *p* = 0.029, respectively), as shown in Figure 4 and summarized in Table S3. An enhancement of lysophosphatidylinositol (LPI) 18:1 was found when comparing AD and control neuronal EVs (*p* = 0.047, Table S3), whereas a decrease in LPI 16:0, LPI 18:0 and LPI 18:1 was found when comparing either neuronal EVs and multi-origin EVs (*p* = 0.013, *p* = 0.002, *p* = 0.003, respectively) or astrocyte EVs and multi-origin EVs of AD samples (*p* = 0.003, *p* = 0.003, *p* = 0.014, respectively). Some of lysophosphatidylserine (LPS) lipids were also significantly changed, such as LPS 18:0 when comparing AD neuronal and astrocytic EVs (*p* = 0.034, Figure 4E), LPS 20:4 when comparing astrocytic AD and control samples (*p* = 0.037, Figure 4F) and LPS 16:0 when comparing neuronal and astrocytic EVs of control samples (*p* = 0.025), as shown in Table S3.

### 3.6. Multivariate PCA

The multivariate analysis provided a holistic view of the variability between EVs isolated from AD patients and healthy controls. In the multi-origin EV samples, the first three PCs accounted for 57% of the sample variability. Clustering of AD and control samples along PC3 is significant, with free cholesterol (FC), PA, LPI, PC, AC and PS lipid species contributing to this separation (Figure 5a,b). Influential individual factors for PC3 include PA with 42:7, 42:5, 30:0, 40:7 and 38:4 fatty acid chains; PI with 38:5, 40:7 and 36:3 fatty acid chains; CE with 18:2, 22:5 and 20:2 fatty acid chains; and LPI with 18:0 and 16:0 fatty acid chains, all with a higher presence in AD all-cell-origin EV samples (negative loadings) (Figure 5f). FC was highly enriched in control samples (positive loadings) (Figure 5f). Additionally, the impact of PA and PS species in driving separation of AD samples from control patients via enrichment in AD samples aligns with univariate comparisons in Figure 1c,e. No significant separation was seen along PC1 or PC2 (Figure 5a). Of the unique lipids in multi-origin EV samples with significantly different concentrations between AD and control subjects, PA species (30:0, 34:0, 38:4, 40:4, 40:7) and PS species (36:0, 36:2, 38:1, 38:2, 38:3, 38:4, 38:5, 40:5, 40:6) were the most abundant and all enriched in AD subjects (Figure 8a). Cer d18:1/18:0, LacCer d18:0/20:0, PCe 36:0, 36:1, PI 42:6 and LPS 20:1, 24:1 were also enriched in the AD group (Figure 9a). BMP 32:1, PG 36:0, and AcylPG 16:0/36:0 were significantly depleted in AD samples (Figure 8a). Given the low N, no individual lipid species remained significant after multiple-testing (Bonferroni) correction.

In AEVs, 56% of the variation was explained in PC1, PC2 and PC5 collectively. Significant separation between AD and control AEV samples was only seen in PC5, with the PI, PS and GM3 mainly contributing to group separation in the positive direction along this component and mainly FC, DG and LPS contributing to separation in the negative direction (Figure 6b). Individual lipids with large, positive loadings include PI with 32:0, 40:6, 34:0, 36:3, 40:5, 36:2, 38:2, 36:0 and 36:1 fatty acid chains; PS with 38:4, 38:1, 38:3, 38:2, 36:1, 38:5 and 36:0 fatty acid chains; and GM3 with d18:1/18:0, d18:0/16:0 and d18:1/18:1 fatty acid chain pairs (Figure 6f). LPCe with 20:2 and 20:1 fatty acid chains and Cer d18:1/22:1 had large loadings in the negative direction of PC5 (Figure 6f). No significant separation was seen along PC1 or PC2 (Figure 6a). The lipid profiles of AEV samples from AD and control subjects contained significant differences in concentrations of PS species (36:1, 36:2, 38:1, 38:4), all enriched in AD patients (Figure 8b). These findings align with univariate analysis comparing AD and control groups in AEVs (Figure 1e). PI 38:5, dhCer d18:0/20:0, PG 30:0, BMP 38:1, 38:4 and LPE 20:1 were also enriched in AEVs from AD subjects, while PE 36:0, PEp 38:6, 40:7 and DG 40:5/18:0 were depleted (Figure 8b). Of these species, PG 30:0 remained significant after multiple-testing (Bonferroni) correction.

In the PCA for NEVs, 59% of the variation is explained by the first three PCs (Figure 7a–c). No significant clustering between AD and control NEV samples was observed in any of the PCs. The largest distinction between the two groups is seen along PC3 (*p* = 0.06) (Figure 8a–c). The GM3, NSer and NAPE classes of lipids on average contribute most to the variability along PC3 (Figure 7a–c). Of the individual species with large absolute loadings, PCe with 38:6, 38:1, 38:2 and 38:0 fatty acid chains and MG with 22:0, 20:1, 22:2 and 20:3 fatty acid chains had positive loadings (in the direction of the control NEV samples) and PC with 42:7 and 42:6 fatty acid chains; AcylPG with 16:0/36:4, 16:0/38:4 and 16:0/36:0 fatty acid chain pairs; and NAPE with p18:1/20:4/20:4 and p18:1/22:4/20:4 fatty acid chains had negative loadings (in the direction of the AD NEV samples) (Figure 7d–f).

Of the individual lipids that are significantly enriched in NEVs from AD subjects, GM3 (d18:0/18:0, d18:0/20:0, d18:1/18:0, d18:1/18:1, d18:1/20:0, d18:1/20:1) is the most represented. Additionally, dhSM d18:0/20:1, MhCer d18:0/26:1, Sulf d18:1/20:1, AcylPG 16:0-38:5 and NAPE p18:0/22:4/20:4 are also enriched in NEVs from AD subjects. Only PG 30:0 is significantly depleted in NEVs from AD subjects (Figure 8c). However, none of these lipids remained significant post multiple-testing (Bonferroni) correction. Enrichments for all other individual lipids are outlined in Table S4.

Multivariate analysis was also conducted in AD and healthy controls to characterize the lipid profiles of NEVs and AEVs in comparison to the multi-origin EV population (marked as Tetraspanin-EVs). Together, PC1, PC2 and PC4 accounted for 56% of the variability in EV samples from healthy controls. Both the NEV and AEV subsets separated significantly in the same direction from multi-origin EVs along PC2 and PC4 (Figure 9b,c). Lipid species that heavily contributed to this separation include LPC, LPCe, MhCer and GM3, enriched in multi-origin EVSs, and LPS, enriched in NEVs and AEVs (Figure 9b,c). Additionally the significant impact of LPC species in differentiating multi-origin EVs from brain-derived EVs lines up with the univariate comparisons depicted in Figure 1G. Along PC2, GM3 d18:0/24:0, d18:1/24:0, d18:0/18:0, d18:1/20:0. 818:1/18/1 and d18:0/20:0 and LPCe 18:0, 16:0 and 16:1 had large loadings in the direction of the multi-origin EV cluster (Figure 9e). Along PC4, PEp lipids (38:4, 38:5, 36:4, 34:2) had large loadings in the direction of the NEV clusters, while MhCer lipids (d18:1/20:0, d18:1/22:0, d18:0/24:1, d18:0/22:0, d18:1/24:1, d18:0/24:0) and GB3 lipids (d18:1/22:1, d18:1/24:1, d18:1/26:0) had large loadings in the direction of the Tetraspanin-EV cluster (Figure 9f).

Univariate analysis comparing lipid compositions between EV subsets was also performed. MG lipids (18:2, 20:1, 20:2 and 22:1), DG lipids (32:2/16:1, 36:3/18:1, 38:2/18:1, 38:4/18:1 and 40:5/18:1) and 30 individual TG lipids were significantly enriched in both L1CAM+ and GLAST+ EVs (Figure 10a,b). Of the lipids uniquely enriched in L1CAM+ NEVs when compared to Tetraspanin-EVs, PA lipids (34:1, 34:2, 36:0, 36:1, 36:2, 38:0, 38:1, 38:3, 38:5 and 38:6) and PCe lipids (34:1, 34:2, 36:2, 36:3, 38:5 and 38:6) were highly represented (Figure 10a). Lipid species that were broadly depleted in both NEV subtypes EVs included Sulf (d18:0/16:0, d18:0/18:0, d18:0/24:0, d18:1/16:0, d18:1/16:1, d18:1/18:1, d18:1/20:1, d18:1/22:0, d18:1/22:1, d18:1/24:0 and d18:1/24:1), GM3 (d18:0/16:0, d18:1/16:0, d18:0/18:0, d18:0/20:0, d18:0/22:0, d18:1/18:0, d18:1/18:1, d18:1/20:0, d18:1/20:1, d18:1/22:0 and d18:1/22:1), PG (34:2, 38:0, 38:2, 38:3 and 38:5) and LPI (16:0, 18:0 and 18:1) (Figure 1, Figure 2, Figure 3, Figure 4, Figure 5 and Figure 6a,b). Lipid species that were uniquely depleted in GLAST+ AEVs included MhCer (d18:0/16:0, d18:0/22:0, d18:0/22:1, d18:0/24:0, d18:0/24:1, d18:1/16:0, d18:1/16:1, d18:1/20:0, d18:1/22:0 and d18:1/24:0), dhSM (d18:0/18:0, d18:0/18:1, d18:0/20:0, d18:0/22:0, d18:0/22:1, d18:0/24:0 and d18:0/24:1), LPC (16:0, 16:1, 18:0, 18:1, 20:0, 20:1, 20:2, 20:3 and 20:4) and LPCe (16:0, 16:1, 18:0, 18:1, 20:0 and 21:0) (Figure 6b). When comparing NEVs and AEVs, MhCer lipids (d18:0/24:0, d18:0/24:1, d18:1/20:0, d18:1/22:0 and d18:1/24:1), PC (30:0, 32:0, 32:1, 38:1, 40:7 and 42:7), PCe (30:0, 32:0, 32:1, 40:5 and 42:6) and 11 unique TG lipids were enriched in NEVs, while relatively few lipids were enriched in AEVs (Figure 10c). However, multiple testing-adjusted lipid compositions were not significantly different between EV subtypes. Overall, both univariate differences and holistic multivariate analysis show similarities between AEVs and NEVs that are not present in multi-origin EVs. Enrichments for all other individual lipids are outlined in Table S5.

Analysis of EV subtypes in AD patients depicted lipid profiles distinct from that of healthy controls. Together, PC1, PC3 and PC4 accounted for 46% of the variability in EV samples from AD patients. Multivariate analysis demonstrated significant separation of both NDEV and ADEV subtypes from Tetraspanin-EVs in the same direction along PC3. TG, PS and DG lipid species heavily contributed to this separation, both in the direction of NEV and AEV clusters (Figure 11a,b). Enrichment of DG species in AEVs and NEVs and enrichment of PG in multi-origin EVs from AD patients also aligns with the significant univariate difference in the percent of total lipids, depicted in Figure 11b,f. When looking at the top 25 individual lipids with the largest absolute loadings along PC3, 20 of them are TG species. We also see multiple PS lipids (38:2, 38:4 and 36:1) and DG 34:2/16:0 driving this separation (Figure 11e).

The results from univariate analysis of EVs from AD patients highlighted significant differences in individual lipid concentrations between EV subtypes. Though the lipid composition of EV subtypes varied significantly, only PA 30:0 and LPI 18:0 were significantly enriched in multi-origin Tetraspanin-EVs compared to L1CAM+ NEVs after correction for multiple testing, while PEp 38:4 and DG 40:5/18:1 were significantly enriched in L1CAM+ NEVs compared to multi-origin Tetraspanin-EVs after correction for multiple testing (Figure 12a). Enrichments for all other individual lipids are outlined in Table S5.

## 4. Conclusions

Lipidomics holds substantial promise in investigating the etiology of AD and pinpointing biomarkers for early diagnosis, as perturbed lipid metabolism is linked to disease pathophysiology. This study reports an optimized workflow for EV isolation followed by targeted lipidomic analysis of distinct EV subpopulations (neuronal, astrocytic, multi-origin) in healthy control and AD patients. Significant differences were observed in the levels of lipids in GL, GPL, SL and LPL classes among neuronal, astrocytic and multi-origin EV subtypes, validating their differential derivation from the respective cells. Importantly, more differences were seen when comparing AEVs or NEVs to multi-origin EVs rather than when comparing them to each other. The relative similarity in the AEV and NEV lipidome is consistent with their derivation by developmentally related cell types that reside in the same organ, the brain. These results provided new insights into the role of MS-based lipidomic methodology used to explore plasma EVs. Further investigations encompassing an expanded cohort of AD patients will ascertain whether the lipid constitution derived from subfractions of EVs can be employed as viable tools for diagnostic and prognostic assessment of disease activity.

The present exploratory study suggests that, in NEVs derived from AD samples, PG levels were significantly decreased compared to controls. In NEVs, dhCer levels were significantly diminished compared to AEVs, as well as DG, GM3 and LPI levels compared to multi-origin EVs. On the other hand, PE and PEp were increased in NEVs compared to multi-origin EV samples. Moreover, in AEVs, raised levels of PS were observed in AD compared to control samples, while a decrease in PG, LPC and LPE and an enhancement of MG were found in AEVs derived from AD samples compared to multi-origin EVs. Finally, in multi-origin EVs from AD compared to control samples, PA and PS were significantly increased.

Comparing individual lipid classes in distinct EV fractions derived from AD samples, important insights were provided. MG 20:0 was significantly decreased in AEVs compared to NEVs and multi-origin EVs. SM d18:1/16:0 and SM d18:1/16:1, Cer d18:1/16:1, Cer d18:1/20:1 and Cer d18:1/24:1 as well as Sulf d18:1/18:0, d18:1/24:0 and d18:1/24 were significantly decreased in NEVs compared to multi-origin EVs. Moreover, increased levels of PA 30:0 and decreased levels of PA 40:6 were detected between neuronal and astrocytic EVs. Lastly, LPE 18:0 was reduced in astrocytic EVs compared to multi-origin EVs and LPS 18:0 levels were decreased in neuronal EVs compared to astrocytic EVs.

Between AD and control samples, MG 20:3 and MG 22:2 levels were significantly raised in multi-origin EVs, while DG 40:5/18:0 was reduced in AEVs. PC and PE species were the most enriched classes of GLPs, with significant alterations between AD and control samples in both neuronal and astrocytic EVs. Furthermore, PG 36:0 was increased and LPE 20:2 was diminished in NEVs. Higher levels of PI 40:4 were observed in NEVs and LPS 20:4 in AEVs.

The main strengths of this study are the demanding and multi-step isolation of EVs after depleting lipoproteins, which increases our confidence in attributing differences to the brain, and the derivation of multiple EV types from the same plasma aliquots, which allowed the multi-cell-origin EVs to serve as a control population for AEVs and NEVs. The main weakness of this study is the small number of samples, as a result of the stringent isolation methodology. The negative selection procedure removed predominantly apoA1- and apoE-containing lipoproteins, but may not have efficiently removed apoB-containing lipoproteins. A further limitation is that this small cohort could not adequately reflect the ethnic and racial diversity of the U.S. population at large, let alone the global population. Future studies should expand upon our findings and study larger groups with more diverse backgrounds. The findings of this exploratory study warrant validation in larger cohorts, but already motivate intriguing hypotheses on the role of lipids in AD pathogenesis and highlight certain lipids as candidate biomarkers. As common to all cross-sectional biomarker studies, the findings of this study cannot be used to infer causality. The observed lipidomic differences between individuals with AD and controls may be the result of AD pathogenic changes or causatively implicated in disease pathogenesis. This conundrum may only be resolved by basic studies that control all experimental parameters and biomarker studies relying on longitudinal measurements.

## Figures and Tables

**Table 1:** Relative quantitative percentage (%T-lip) of the average of main lipid classes (sterol lipids, glycerolipids, sphingolipids glycerophosholipids and lysophosholipids) obtained from EV subfamilies (neuronal EVs marked as L1CAM, astrocytes EVs marked as GLAST, multi-origin EVs marked as Tetraspanin) of AD and control samples ^1^.

Lipid Categories	Neuronal EVs AD	Neuronal EVs Control	Astrocyte EVs AD	Astrocyte EVs Control	Multi-Origin EVs AD	Multi-Origin EVs Control
Sterol lipids ^2^	84.28 ± 3.24	81.75 ± 6.41	85.29 ± 1.62	83.27 ± 4.79	85.12 ± 0.89	84.29 ± 4.33
Glycerolipids ^3^	4.24 ± 2.15	5.86 ± 4.01	3.71 ± 1.56	5.13 ± 3.61	2.74 ± 0.60	3.76 ± 2.47
Sphingolipids ^4^	3.37 ± 0.85	3.71 ± 0.99	3.69 ± 0.30	3.73 ± 0.29	4.04 ± 0.36	4.11 ± 0.82
Glycerophospholipids ^5^	7.79 ± 1.32	8.32 ± 2.10 *	7.02 ± 0.95	7.54 ± 1.15	7.72 ± 0.51	7.40 ± 1.59
Lysophospholipids ^6^	0.27 ± 0.09	0.30 ± 0.07	0.24 ± 0.05 *	0.28 ± 0.04 **	0.32 ± 0.09	0.36 ± 0.06

^1^ The values are given as mean ± SD (*n* = 5). ^2^ Sterol lipids include FC + CE. ^3^ Glycerolipids are MG + DG + RG. ^4^ Sphingolipids are CEr + dhCer + SM + dhSM + MhCer + sulf + LacCer + GM3 + GB3. ^5^ Glycerophospholipids are PA + PC + PCe + PE + Pep + PS + PI + PG + BMP. ^6^ Lysophospholipids are LPC + LPE + LPI + LPS. Values for each lipid class are presented in Table S1. Statistically significant samples: * (*p* < 0.05), ** (*p* < 0.01); for GLPs (L1CAM-control vs. Tetraspanin-control), for LPLs (GLAST-AD vs. Tetraspanin-AD; GLAST-control vs. Tetraspanin-control, Table S2).

## Data Availability

Data supporting the reported results can be obtained by contacting the senior author, Dimitrios Kapogiannis, at kapogiannisd@mail.nih.gov.

## Supplementary Materials

The following supporting information can be downloaded at: https://www.mdpi.com/article/10.3390/cells13080702/s1, Figure S1: NTA measurements for EV populations; Figure S2: Percentage differences for total sterol lipids, glycerolipids and sphingolipids in EV subfamilies of AD and control samples; Figure S3: Percentage differences for glycerophosholipids in EV subfamilies of AD and control samples; Figure S4: Percentage differences for lysophospholipids and precursors of lipid metabolism in EV subfamilies of AD and control samples; Table S1: Relative quantitative percentage (%T-lip) and *p* values for lipid classes obtained from EV subfamilies (L1CAM, GLAST, Tetraspanin) of AD and healthy control samples; Table S2: The *p* values of the average of main lipid categories (sterol lipids, glycerolipids, sphingolipids and glycerophosholipids) obtained from EV subfamilies (L1CAM, GLAST, Tetraspanin) of AD and control samples; Table S3. Relative quantitative percentage (%T-lip) and *p* values for each lipid class obtained from EV subfamilies (L1CAM, GLAST, Tetraspanin) of AD and healthy control samples; Table S4. Enrichments for all individual lipids in NEVs from AD subjects; Table S5. Enrichments for all individual lipids between EV subsets; Table S6: Concentration and size of three different EV populations.
