# Supplementary material for: Lipidomic Analysis of Plasma Extracellular Vesicles Derived from Alzheimer’s Disease Patients
Source: Cells. 2024 Apr 18;13(8):702. doi: 10.3390/cells13080702 (PMC11049154; doi:10.3390/cells13080702)
Supplement: Supplementary file 1 [file cells-13-00702-s001.zip › Supplemental_Figures.pdf]

Supporting Information

Lipidomic Analysis of Plasma Extracellular Vesicles derived from Alzheimer's disease patients

Marios G. Krokidis, Krishna A. Pucha, Maja Mustapic, Themis P. Exarchos, Panagiotis Vlamos and Dimitrios Kapogiannis*

*Correspondence: kapogiannisd@mail.nih.gov

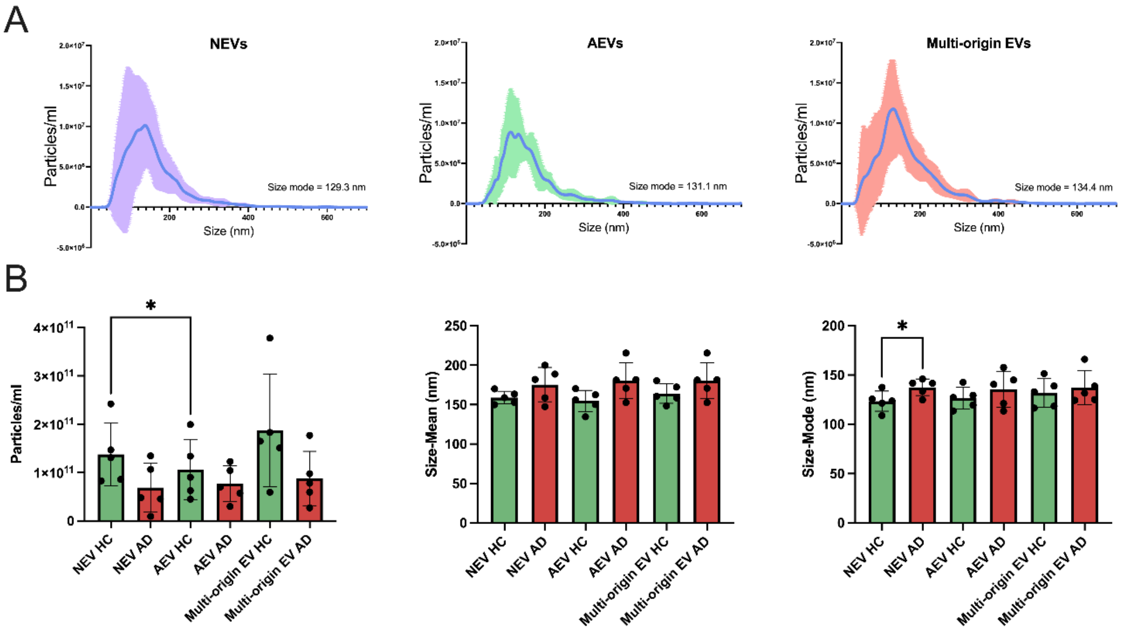

Figure S1. A. NTA measurements showing average ± SD EV of concentration (particles/ml) and size distribution (nm) across three EV populations (NEV, AEV, Multi-origin EV). Each EV population shows combined measurements for AD and HC individuals; N = 10 per graph. B. Graphs depict average concentration and average diameter (mean, mode) for three EV populations (NEV, AEV, Multi-origin EV) and two groups (AD, HC); N= 5 per column. We used paired t-test to assess differences; * $p < 0.05$.

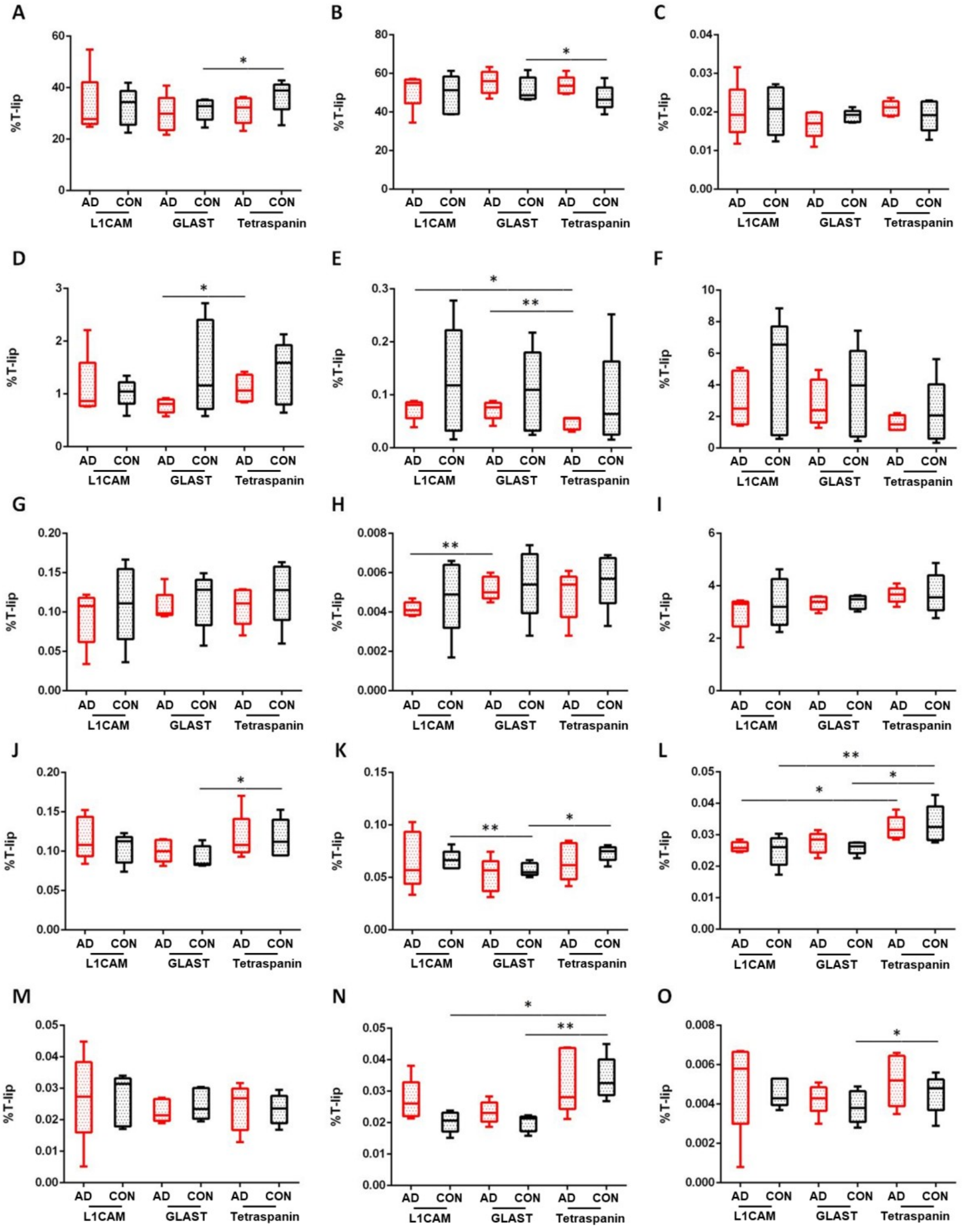

Figure S2. Percentage differences for total sterol lipids (A to B), glycerolipids (D to F) and sphingolipids (G to O) in EV subfamilies (L1, GL, CD) of AD and health control samples. A) Free cholesterol, B) cholesterol ester, C) acyl carnitine, D) monoacylglycerol, E) diacylglycerol, F) triacylglycerol,G) ceramide, H) dihydroceramide, I) sphingomyelin, J) dihydrosphingomyelin, K) monohexosylceramide, L) sulfatide, M) lactosylceramide, N) monosialodihexosylganglioside, O) globotriaosylceramide. L1CAM are neuronal EVs; GLAST are astrocyte EVs; Tetraspanin-EVs are CD81, CD9, CDC3 (surrogate of all cell origin). The values are given as mean ± SD (n = 5). Statistical significances: * ($p < 0.05$), ** ($p < 0.01$).

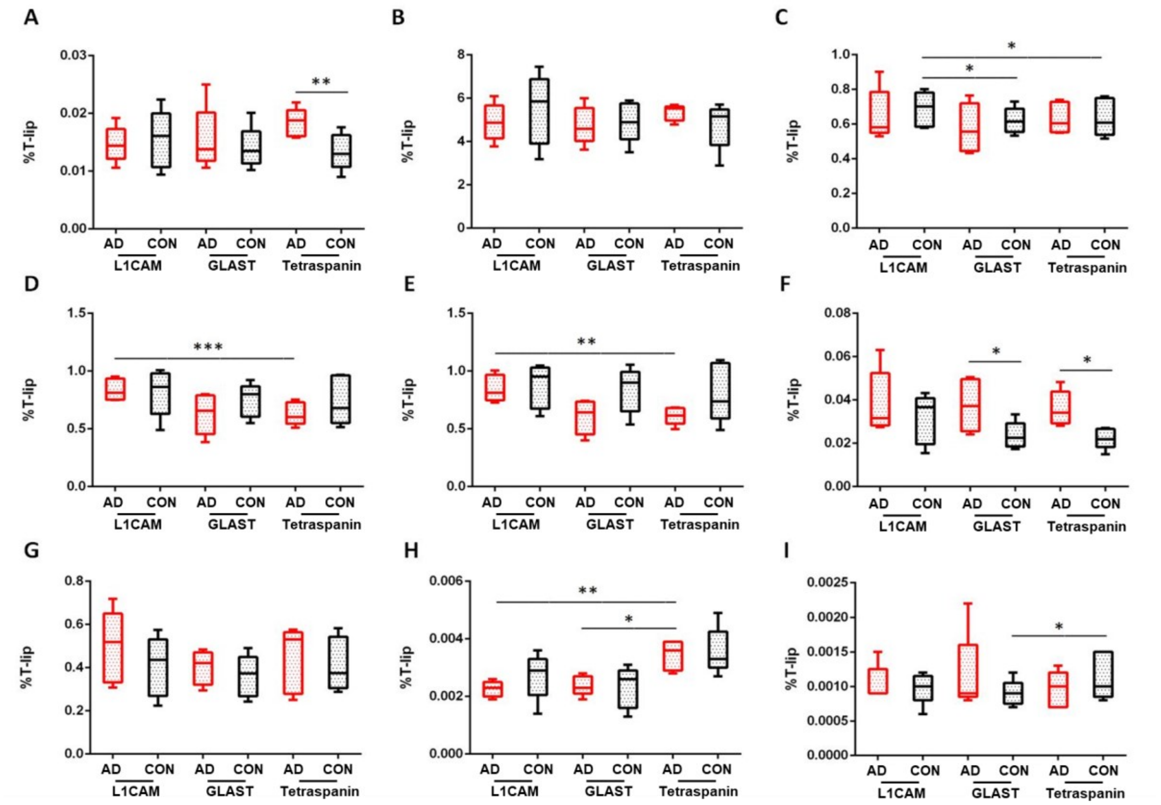

Figure S3. Percentage differences for glycerophosholipids in EV subfamilies (L1, GL, CD) of AD and health control samples. A) Phosphatidic acid, B) phosphatylcholine, C) ether phosphatidylcholine, D) phosphatidylethanolamine, E) plasmalogen phosphatidylethanolamine, F) phosphatidylserine, G) phosphatidylinositol, H) phosphatidylglycerol, I) bis(monoacylglycero)phosphate. The values are given as mean ± SD (n = 5). L1CAM are neuronal EVs; GLAST are astrocyte EVs; Tetraspanin-EVs are CD81, CD9, CDC3 (surrogate of all cell origin). Statistical significances: * ($p < 0.05$), ** ($p < 0.01$), *** ($p < 0.001$).

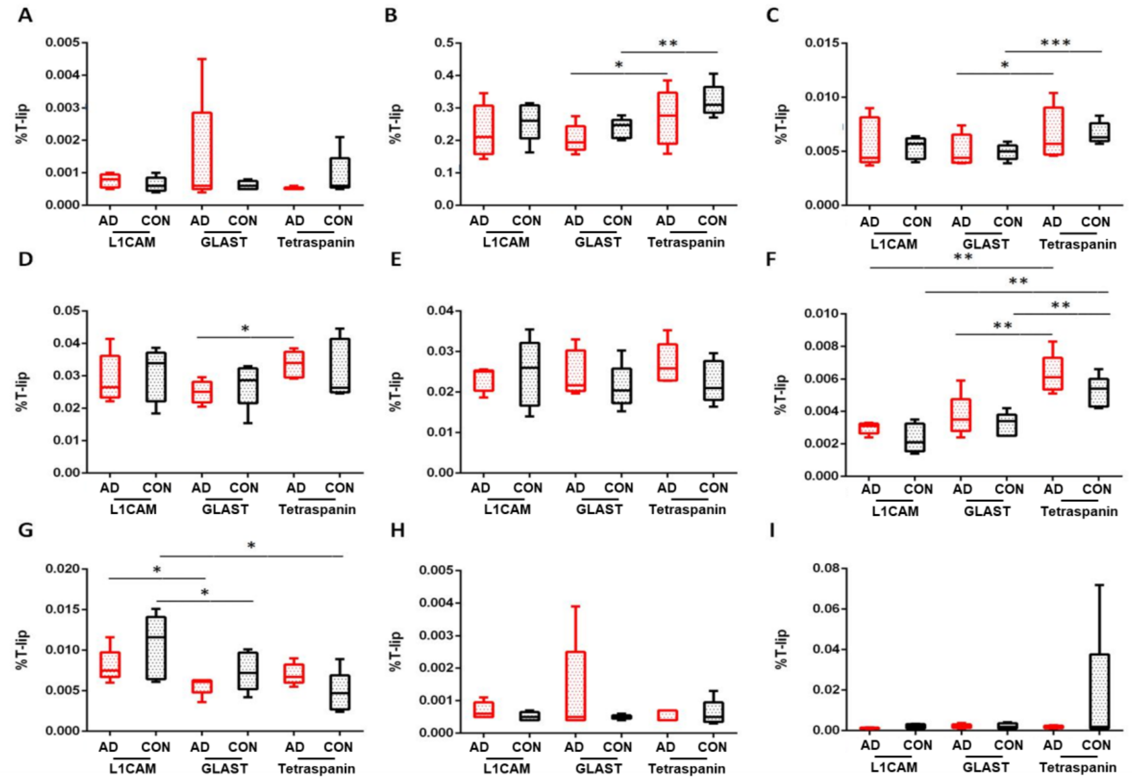

Figure S4. Percentage differences for lysophospholipids and precursors of lipid metabolism in EV subfamilies (L1, GL, CD) of AD and health control samples. A) Acyl phosphatidylglycerol, B) lysophosphatidylcholine, C) ether lysophosphatidylcholine, D) lysophosphatidylethanolamine, E) plasmogen lysophosphatidylethanolamine, F) lysophosphatidylinositol, G) lysophosphatidylserine, H) N-Acyl Phosphatidylethanolamine, I) N-Acyl Serine. L1CAM are neuronal EVs; GLAST are astrocyte EVs; Tetraspanin-EVs are CD81, CD9, CDC3 (surrogate of all cell origin). The values are given as mean ± SD (n = 5). Statistical significances: * ($p < 0.05$), ** ($p < 0.01$), *** ($p < 0.001$).
